# Risk Factors of Kidney Stones in Khyber Pakhtunkhwa, Pakistan: A Descriptive Cross-Sectional Study

**DOI:** 10.7759/cureus.63080

**Published:** 2024-06-24

**Authors:** Muhammad Waqas, Zaryaab A Khan, Shabeer Ahmad, Siddiq Akbar, Nayab Khalid

**Affiliations:** 1 Urology, Hayatabad Medical Complex, Peshawar, PAK; 2 Nephrology, Khyber Teaching Hospital, Peshawar, PAK; 3 General Surgery, Hayatabad Medical Complex, Peshawar, PAK

**Keywords:** renal calculi, pakistan, risk factors, urolithiasis, kidney stones

## Abstract

Introduction

Renal calculi, commonly known as kidney stones, affect approximately 12% of the global population, often resulting in mild to severe pain and complications such as infection and renal failure. The causes are multifactorial, involving lifestyle factors, genetic predisposition, and various medical conditions. Despite advancements in treatment, the incidence of kidney stones is on the rise, especially in regions like Asia's stone belt, which includes Pakistan as well. This study investigates the risk factors associated with kidney stones in patients at the Hayatabad Medical Complex, Peshawar, Pakistan, with the goal of informing healthcare policymakers and educating patients to reduce the prevalence and economic burden of the disease in the region.

Methods

Over a four-month period, 173 patients admitted to the department with confirmed urolithiasis were recruited using a consensus sampling technique. Inclusion criteria were adults aged 18 or older residing in Khyber Pakhtunkhwa, Pakistan. Exclusion criteria included pregnancy, incomplete medical records, secondary causes of renal stones, and unwillingness to participate. Data on socio-demographic factors, clinical history, and lifestyle habits were collected through structured interviews. Descriptive analysis was performed using SPSS software version 23 (IBM Inc, Armonk, New York).

Results

The study findings indicate that kidney stones were notably prevalent among patients aged 31 to 45 years, with 85 cases identified, comprising 49.1% of the study participants. Furthermore, a significant proportion of affected individuals were males, accounting for 94 cases (54.3%). Those with primary schooling or lower education comprised 106 cases (61.3%), and 124 individuals (71.7%) had lower socioeconomic status. Overweight individuals constituted 81 cases (45.8%) of the total. The study also found a considerable prevalence among residents of rural areas, with 128 cases identified (74.0%). Additionally, 104 cases (60.1%) had a personal history of renal stones. Insufficient water intake was prevalent among 122 individuals (70.5%), while daily consumers of more than one cup of tea also exhibited a higher prevalence, with 97 cases identified (56.1%). Lastly, individuals with a high sodium diet were notably affected, with 112 cases (64.7%) recorded.

Conclusion

In summary, individuals aged 31-45, males, those residing in rural areas, with lower educational and socioeconomic status, insufficient water intake, a diet high in sodium, personal history of kidney stones, and overweight are at higher risk of urolithiasis. Public education on preventive measures is essential to decrease the incidence of kidney stones and enhance quality of life.

## Introduction

Kidney stone disease, a condition known for centuries [1], affects approximately 12% of the global population at some point in their lives [2]. Kidney stones, also known as renal calculi, are hard deposits that form in the pelvis of the kidney [3]. However, they typically go unnoticed until they obstruct the renal pelvis or pass through the ureter, causing pain that ranges from mild discomfort to severe waves of pain. These stones can lead to complications such as urinary tract infections, hydronephrosis, pyelonephritis, ureteral stricture or perforation, decreased renal function, and, ultimately, renal failure [4].

The development of kidney stone disease involves multiple factors such as age, gender, level of education, and socioeconomic status. Other contributing elements include insufficient hydration, consumption of soft drinks, obesity, a sedentary lifestyle, family history, smoking, hyperlipidemia, diabetes mellitus, and hypertension [5-7]. The treatment of renal stones varies according to the patient, stone type and size, and location of the stone [8]. Although minimally invasive surgical and non-surgical treatment options are available, they often require additional procedures that prolong treatment, increase patient suffering, and consume valuable healthcare resources [9]. Additionally, some energy sources used for stone disintegration may promote local stone formation or damage surrounding structures.

Even with the improvements in treatment strategies for urinary stones, the global incidence of renal stones continues to increase [2], and approximately half of the patients with kidney stones will undergo a second episode of renal colic within 10 years [10]. In a meta-analysis of 58 studies, Liu et al. reported that the prevalence of kidney stones ranges from 1-19.1% in West Asia, Southeast Asia, South Asia, South Korea, and Japan, and these areas in Asia are referred to as the "Stone Belt". Conversely, in most other parts of East and North Asia, the prevalence of kidney stones is lower, ranging from 1-8% [11].

Due to Pakistan's position in the Stone Belt, it has a high prevalence of kidney stones, with a reported prevalence of 2.8% [12]. Because the high prevalence and lifetime incidence of recurrent renal stones ranges from 10-75% [13], this condition imposes a significant financial burden on society through healthcare expenses and lost productivity. Despite that, there are limited studies available reporting the factors associated with the making of kidney stones in the region, especially in the northwest region of Pakistan.

The study aims to explore the risk factors for kidney stone disease among admitted patients with urolithiasis at the Institute of Kidney Diseases, Hayatabad Medical Complex, Peshawar, Pakistan. This study aims to contribute valuable insights for healthcare professionals, policymakers, and the general public. By laying the groundwork for further investigations, we seek to reduce the incidence and burden of renal stone disease in our region.

The objective of this study was to identify and document the risk factors associated with the development of kidney stones in the population of Khyber Pakhtunkhwa, Pakistan.

## Materials and methods

Following approval from the Institutional Research and Ethical Board (IREB; approval no. 1674), this descriptive cross-sectional study was conducted at the Department of Urology in the Institute of Kidney Diseases, Hayatabad Medical Complex, Peshawar, Pakistan, over a period of four months from January 1st, 2024, to April 30th, 2024.

The study comprised of admitted patients to the Department of Urology with urolithiasis. The sample size was calculated using OpenEpi, version 3, an open-source calculator, with an anticipated frequency of 12% [2], a margin of error of 5%, and a confidence interval of 95%. Recruitment was done using a non-probability sampling technique.

Inclusion criteria comprised all the in-patients during the period of the study duration with a confirmed diagnosis of renal stone disease confirmed with Ultrasound or CT kidney, ureter, bladder (KUB), aged 18 years or older, residents of Khyber Pakhtunkhwa province of Pakistan, and willing to participate in the study. Exclusion criteria were pregnant patients, patients with incomplete medical records or missing crucial information necessary for the study, patients with renal stones due to secondary causes such as metabolic disorders or anatomical abnormalities, and those unwilling to participate. Informed consent was obtained from all participants prior to participation. Data were collected through structured interviews using a self-made questionnaire.

Socio-demographic factors were assessed, including age; gender; body mass index (BMI), which was categorized as normal (18.50-24.90 kg/m²), underweight (<18.50 kg/m²), overweight (25.00-29.90 kg/m²), and obese (≥30.00 kg/m²); level of education (primary or lower, middle, matriculation, intermediate and above); residential area (urban or rural); and socioeconomic status categorized by monthly income (low: ≤40,000 PKR, middle: 40,001-80,000 PKR, upper: >80,000 PKR). Clinical history was also recorded, including personal and family history of renal stones, smoking, hypertension, and diabetes. Lifestyle factors assessed were water intake (sufficient: ≥3 liters daily, insufficient: <3 liters daily), daily consumption of soft drinks, daily consumption of tea of more than 8 ounces, and a diet high in sodium (daily consumption of fast food or intake of more than one teaspoon of added salt daily).

The data were analyzed through SPSS software version 23 (IBM Inc., Armonk, New York). Descriptive analysis was conducted to calculate the frequencies and percentages of qualitative variables, as well as the means of quantitative variables.

## Results

**Figure 1:**
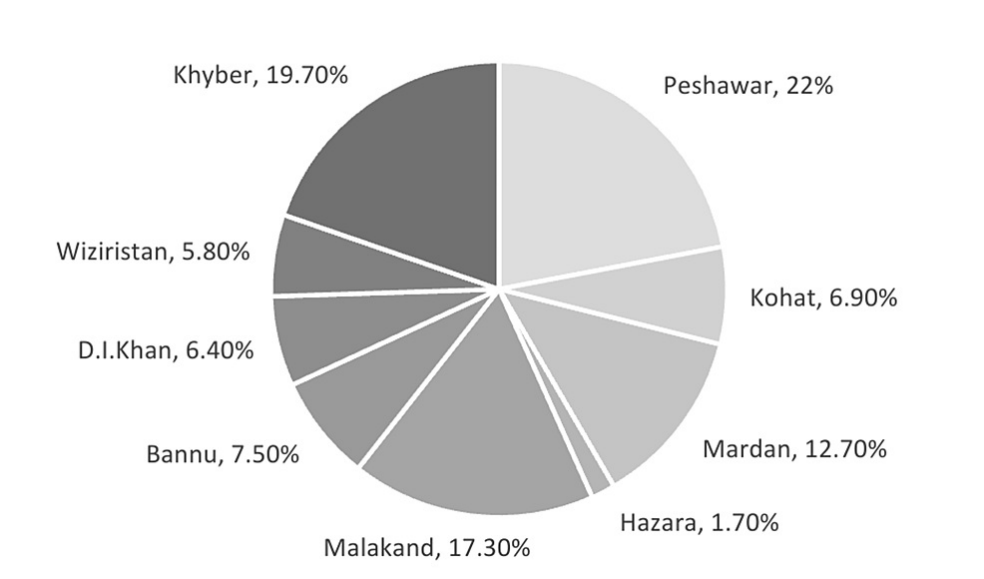
Pie chart depicting the geographical distribution of the study participants

A total of 173 participants were included in the study after setting the inclusion and exclusion criteria. The mean age of study participants was 34.78 years, with a standard deviation (SD) of ±9.89. Among them, 94 (54.3%) were males and 79 (45.7%) were females. The majority of the study participants were residents of Peshawar, followed by the Khyber and Malakand divisions (Figure 1).

Kidney stones were more frequently observed in the male gender, aged 31-45, with lower educational and socioeconomic status. Additionally, a higher incidence was noted among individuals with a BMI falling within the normal and overweight ranges, as well as those residing in rural areas (Table 1).

**Table 1:** Sociodemographic information of the participants

Variable	Frequency (n)	Percentage (%)
Gender		
Male	94	54.3
Female	79	45.7
Age bands in years		
Below 30	63	36.4
31 to 45	85	49.1
Above 45	25	14.5
Body mass index (BMI)		
Underweight	6	3.5
Normal	78	45.1
Overweight	81	45.8
Obese	8	4.6
Residential area		
Urban	45	26.0
Rural	128	74.0
Socioeconomic status		
Low	124	71.7
Middle	38	22.0
Upper	11	6.4
Educational status		
Primary or lower	106	61.3
Middle	32	18.5
Matriculation	21	12.1
Intermediate or above	14	8.1

Table 2 shows the clinical history of the study participants. The occurrence of renal stones was higher among patients with a personal history of renal stones, no family history of renal stones, no diabetes mellitus, or hypertension compared to those without a personal history of stones, with a family history of renal stones, diabetes mellitus, or hypertension, respectively. Nevertheless, a notable proportion of patients with renal calculi did have a family history.

**Table 2:** Clinical history recorded for the participants

Variable	Frequency (n)	Percentage (%)
Family history		
Yes	75	43.4
No	98	56.6
Personal history		
Yes	104	60.1
No	69	39.9
Hypertension		
Yes	23	13.3
No	150	86.7
Diabetes		
Yes	14	8.1
No	159	91.9
Smoking		
Yes	41	23.7
No	132	76.3

Patients with an insufficient intake of water, who consumed more than one cup of tea daily, and a diet high in sodium were found to have a high frequency of kidney stone disease (Table 3).

**Table 3:** Lifestyle habits observed among the study participants

Variable	Frequency (n)	Percentage (%)
Daily water intake		
Sufficient	51	29.5
Insufficient	122	70.5
Daily tea consumption		
One cup (8oz) or less	76	43.9
More than one cup (>8oz)	97	56.1
Daily soft drink consumption		
Yes	44	25.4
No	129	74.6
High sodium diet		
Yes	112	64.7
No	61	35.3

## Discussion

This cross-sectional study, conducted over four months, investigates the risk factors contributing to renal calculi development among adult patients admitted to the Department of Urology at Hayatabad Medical Complex in Peshawar, Pakistan. The study indicates a higher prevalence of kidney stones among males (53.3%) compared to females (45.7%). This is consistent with findings from studies conducted in other regions of Pakistan, including Rawalpindi (56.65%), Lahore (54%), and Karachi (62.3%) [14-16]. Kidney stones were notably more prevalent (49.1%) among individuals aged 31 to 45 years, followed by the age group of 18 to 30 years (36.4%). Similarly, studies in Karachi and Multan also reported a high incidence of kidney stones among participants aged 30 to 49 years, at frequencies of 61.1% and 53.6%, respectively [16,17]. However, studies conducted in Lahore and Rawalpindi indicated that kidney stones were more prevalent (47.55%) among those aged 30 years or below, at rates of 47.55% and 54%, respectively, followed by the age group of 31 to 45 years (28.68%) and 31 to 40 years (34%), respectively [14,15]. Our study revealed an increased incidence of renal stones among individuals with normal BMI and those classified as overweight, as well as among those with a lower socioeconomic and educational status and residing in rural areas. These trends are consistent with observations in other studies [14,15].

The prevalence of renal stones was higher in individuals with a previous history of stones (60.1%), whereas only 43.4% of those with a family history experienced kidney stone disease, and 56.6% of patients with no family history developed stones. Previous research in Pakistan indicated a significant incidence of renal stones in individuals with a positive family history [14,17]. Conversely, a study in India yielded findings consistent with our own, showing that only 23.12% of individuals with renal stones had a positive family history, while 43.24% had a positive personal history [18]. In our study population, renal stones were found to be more prevalent among individuals who were normotensive, non-diabetic, and non-smokers. This aligns with findings from previous studies conducted in other regions of Pakistan and India [14,15,18].

This study revealed that kidney stones are more frequent in individuals with insufficient intake of water (70.5%), daily consumption of tea more than 8oz (56.1%), and in those who consume a diet high in sodium. A study in Rawalpindi showed an increased incidence of kidney stones (61.53%) in individuals whose daily intake of water is less than three liters [14]. Similarly, a study conducted in Lahore reported an increased incidence of stones in those who daily consume soft drinks (55%) and tea (60%) [15]. In a study conducted in India, a significant association was found between a high intake of sodium and tea, but no significant relationship between soft drink consumption and kidney stones was observed [18].

The cross-sectional design of this study provided valuable insights into local risk factors for kidney stone disease, and its findings hold direct relevance to the local community, contributing to the understanding of the risk factors associated with the development of kidney stone disease within this specific population. However, its cross-sectional design and the absence of a case-control design limit identifying associations between variables, emphasizing the necessity for further research. Therefore, further research employing different study designs conducted in diverse geographic areas is important to understand these factors associated with renal stone development and to identify potential variations in risk factors across different regions.

## Conclusions

The findings of our study indicate a higher prevalence of kidney stones among males aged 31-45, those residing in rural areas, with lower educational and socioeconomic status. Key risk factors identified include insufficient fluid intake, high tea consumption, personal history of renal stones, high sodium diet, and elevated BMI. A notable proportion of patients lacked diabetes mellitus, hypertension, and smoking history. While a positive family history of renal stones was not predominant, a significant number of study participants had a family history of renal stones, hence emphasizing the role of genetic predisposition. We advocate for healthcare systems to educate the public on these risk factors and preventive measures to reduce incidence rates, thereby improving the overall quality of life and preventing complications associated with kidney stones.
